# Factor structure of the Young Positive Schema Questionnaire in an eating disorder sample

**DOI:** 10.1007/s40519-023-01549-0

**Published:** 2023-02-17

**Authors:** Tyrone J. Huckstepp, Andrew Allen, Anthea L. Maher, Catherine Houlihan, Jonathan Mason

**Affiliations:** 1 The Thompson Institute, University of the Sunshine Coast, Birtinya, QLD Australia; 2 Discipline of Psychology, School of Health, University of the Sunshine Coast, Sunshine Coast, Australia; 3 Wandi Nerida, Residential Eating Disorders Facility, Mooloolah Valley, Sunshine Coast, QLD Australia; 4 Cairnmillar Institute, Hawthorn East, VIC Australia

**Keywords:** Eating disorders, Factor structure, Psychometric, Schema

## Abstract

**Purpose:**

The Young Positive Schema Questionnaire (YPSQ) measures early adaptive schemas (EAS) which could be used to develop positive psychology and schema-based interventions to benefit the treatment of eating disorders (EDs).

**Methods:**

The present study investigated the factor structure of the YPSQ in a sample of 826 participants (18–73 years; *n* = 753 women) with ED symptomatology (e.g., restricting, binging, and purging). The sample was randomly split into two groups for exploratory and confirmatory factor analyses. Full sample analysis using Pearson correlations was conducted to explore convergent validity of the new YSPQ factor structure with ED symptomatology, emotional regulation, and cognitive flexibility.

**Results:**

A nine-factor model was found, demonstrating good fit indices and internal consistency (α = 0.77–0.92). The YPSQ showed an inverse relationship to ED symptomatology and emotional suppression, and a positive relationship with cognitive flexibility and emotion reappraisal.

**Conclusion:**

Further research is needed to explore the clinical benefits of the YPSQ to identify EAS deficits in individuals with EDs to improve treatment outcomes.

**Level of evidence:**

Level V, descriptive study.

## Introduction

Eating disorders (EDs) account for some of the highest fatality rates of all mental disorders, despite calls to improve service delivery and efficacy of treatment interventions [4, 9]. The lifetime prevalence of anorexia nervosa, bulimia nervosa, and binge eating disorder in the general population of adolescents and adults, worldwide, is 2.8%, 1.5%, and 2.3% in women and 0.3%, 0.1%, and 0.3% in men [31]. EDs are considered one of the most difficult psychological disorders to treat due to their physical and psychological complexity, and sometimes chronic trajectory [41]. Given the impact of EDs, several theoretical models have been applied to understand their nature and inform treatment approaches (e.g., medical, pharmacological, and psychological). In adults, Fairburn’s transdiagnostic theory [27] is the leading model in the field of ED treatment, using enhanced cognitive behavioural therapy (CBT-E) as a primary intervention approach. However, only 40–60% of individuals with an ED diagnosis achieve full recovery after treatment and one-third of individuals do not receive treatment at all [26, 45, 52, 54]. Such findings indicate that modifications to current treatment practices or exploration of new and novel treatment approaches are needed to improve clinical outcomes and treatment retention rates for individuals with EDs.

Several factors have been identified that contribute to ED treatment difficulties, such as the egosyntonic nature of the illness often associated with anorexia nervosa, and the quality of the therapeutic relationship [1, 86]. Problematic behaviours (e.g., body checking, restricting, binging, and purging) and underlying beliefs (e.g., perfectionism and overevaluation of self-worth based on shape and weight) that maintain EDs often serve the function of avoiding negative emotions and can overlap with the individual’s values [32]. Therefore, interventions that focus on addressing maintaining factors can be seen as a threat to the individual’s identity or the removal of a coping mechanism [32]. Furthermore, individuals with EDs report being satisfied with treatment when they feel accepted, cared for, and valued [35] and less satisfied when therapy is focused solely on behavioural goals (i.e., targeting maintaining factor) and neglecting core psychological, emotional, and relationship issues [28]. There are intrinsic challenges with developing a positive therapeutic relationship with individuals with EDs given the complex nature of the disorder (e.g., high prevalence of childhood maltreatment [61], and high co-morbidity of personality disorders [59]). It is not uncommon for clinicians to experience transference and countertransference with individuals with EDs, particularly those with dysregulated personality styles or a history of trauma, sexual abuse, or self-harm [12]. Common countertransference responses from clinicians are anger/frustration, incompetence, boredom, and hopelessness [74, 85], which could maintain the individuals’ maladaptive self-beliefs.

The main component of CBT-E (i.e., stage-three; [27]) focuses on addressing the maintaining factors of EDs. This approach is useful for challenging cognitions and modifying behaviours but may lack depth when addressing childhood experiences and the development of maladaptive beliefs which are core element of ED pathology [60, 68]. Schema therapy has the potential to address the limitations of CBT-E and the previously mentioned factors that contribute to ED treatment difficulty, due to the emphasis of the importance of the therapeutic relationship and emotional experience linked to early life experiences [68, 78].

### Schema therapy

Schema therapy is an integrated cognitive-behavioural and emotion-focused model that links psychological presentations to childhood experiences [94]. The underlying concept of schema therapy proposes that experiences in early childhood and adolescence shape the way an individual views the world and how they adapt strategies to cope with unpleasant emotions and experiences. Furthermore, the model suggests that the development of Early Maladaptive Schemas (EMS) may result when a child’s core emotional needs are not adequately met [96]. The Young Schema Questionnaire (YSQ; [94]) was developed to measure maladaptive schemas of people with personality disorders, through a self-report questionnaire. Now in its third revision [95], the YSQ has been validated in a variety of countries (e.g., Australia and Korea [5], Germany [47], Iran [71], and Turkey [80]), age groups (e.g., older adults [67],and adolescents [73]), and clinical populations [39, 49, 70] showing moderate to good internal consistency (i.e., α = 0.63 to 0.85). The YSQ has been used extensively in ED populations, as individuals with EDs often present with complex maladapted beliefs. Individuals with EDs have significantly higher levels of all EMS, except entitlement, compared to healthy populations, with research being the most consistent for showing higher scores for abandonment, defectiveness, social isolation, self-sacrifice, and unrelenting standards [2, 13, 21, 46, 50, 51, 58, 89–91]. Although preliminary evidence supports the conceptual application of schema therapy for EDs, ongoing research has highlighted the potential benefit of strength-based approaches in formulations of mental disorders [75].

### Positive psychology

Individuals with EDs are more likely to be sensitive to rejection and perceived criticism from others [19]. As such, a positive, strength-based approach may be beneficial in strengthening the therapeutic alliance, improving self-esteem, preventing activation of EMS, and reducing possible elevation bias derived from negatively worded psychometric measures [76]. A therapeutic relationship focused on acceptance, care, and unconditional positive regards could result in improved treatment outcomes and reduced therapy dropout [35]. Two studies by Enrique et al. [22] and Harrison et al. [38] that applied positive psychology principles to individuals with EDs have shown promising results. Results showed that individuals with an ED diagnosis experienced sustained improvements in positive affect and life satisfaction three-to-six months after the implementation of a four-to-five-week positive psychology intervention. However, additional research is needed to further establish positive psychology approaches for ED interventions. One such approach has been made by Louis et al. [57] who developed and validated a 56-item Young Positive Schema Questionnaire (YPSQ) to compliment the YSQ, thus integrating positive clinical psychology and schema theory.

### Young Positive Schema Questionnaire

The YPSQ assesses 14 early adaptive schemas (EAS) which have each demonstrated weak to strong negative correlations with their EMS counterpart (i.e., *r* = 0.22–0.72; [57]. Louis et al. concluded that the EAS are not the counter opposites of EMS and both factors should be taken into consideration. The YPSQ was initially validated in a community sample, which limits its application to clinical populations. However, a recent paper by Paetsch et al. [63] attempted to address this shortcoming by validating a German translated version of the YPSQ in a German sample of 1418 community members and 182 psychiatric patients. An exploratory factor analysis was used in half the community sample and a confirmatory factor analysis in both the second half of the community sample and the psychiatric patient sample. The results found a reduced 41-item, 10 factor structure, with adequate model fit in both the community sample and the psychiatric patient sample. The internal consistency for all factors across both the community sample and the psychiatric sample were good, with an average Cronbach’s alpha of 0.83 (Cronbach’s α = 0.70–0.90) and 0.81 (Cronbach’s α = 0.74–0.91), respectively [63]. Although Paetsch et al. addressed some of the gaps in the literature, several gaps remain that require additional exploration for further development of the YPSQ, such as: (1 The factor structure was determined in the community sample, which may differ when explored in a sample with ED symptomatology, and (2 The psychiatric patient sample was defined as having a diagnosis of major depressive disorder which may not accurately represent individuals with other types of mental health symptomatology. To date, no research has explored EAS in individuals with ED symptomatology. Examination of EAS in EDs could contribute to further conceptualisation and understanding of conditions featuring disordered eating behaviour and may aid in future development of schema-based therapeutic interventions that are underpinned by schema theory and positive psychology.

### Present study

This study explored the psychometric properties and factor structure of the YPSQ in a cross-national English-speaking population of individuals with ED symptomatology and compared it with results from Louis et al. [57] and Paetsch et al. [63]. The study also examined the construct validity of the YPSQ by comparing EAS to ED symptomatology, emotional regulation, and cognitive flexibility. Previous research has shown that individuals with EDs are more likely to suppress their emotions, less likely to use cognitive reappraisal to manage their emotions [16, 18], and have poorer cognitive flexibility compared to individuals without EDs [65, 83, 84]. It was hypothesised that (1) a reduced YPSQ structure would emerge compared to that of the initial 14 factor/EAS structure of the validation study by Louis et al. [57], and would be more similar to that of Paetsch et al. [63] due to the similarities in clinical characteristics of the psychiatric patient sample, and (2) the YPSQ would show a significant inverse relationship to all Eating Disorder Examination Questionnaire (EDE-Q) subscales and the Expressive Submission subscale of the Emotional Regulation Questionnaire (ERQ), while showing a significant positive relationship with the Cognitive Reappraisal subscale of the ERQ and the total score and both subscales of the Cognitive Flexibility Inventory (CFI).

## Methods

### Participants

Participants were included in the study if they were ≥ 18 years and met a clinical cut-off score for the EDE-Q [24] based on previous research by Mond et al. [62] and Simpson et al. [79]. Participants were identified to have significant ED symptoms if their EDE-Q Global cut-off score of was ≥ 2.3 in addition to meeting one of two criteria (as per Mond et al.), being, repeated objective binging episodes (defined as eating what other people would regard as an unusually large amount of food, given the circumstances, and a sense of having lost control over your eating) and/or compulsive exercise as a way of controlling weight or shape, occurring at least four times over the last 28 days [62]

A total of 2426 individuals responded to the survey with 1420 complete responses having no missing data. Of the 1006 incomplete responses, 156 included at least the YPSQ, which was the first scale after the demographics, and 67 of these responses included the EDE-Q. Thus, a total 1487 participants were included in the analysis. The other 850 incomplete responses were excluded due to either not giving consent, not meeting the minimum age criteria, or ending the survey before the first YPSQ (i.e., having at maximum only completed the demographic items). Of the remaining 1487 participants, 826 met the inclusion criteria. The final sample consisted of 753 women (91.2%) and 60 men (7.3%), between the ages of 18 to 73 years (*M* = 26.8, SD = 8.6). The majority of participants were North American (51.3%), Australian (14.5%), and British (9.3%). Almost half (47.3%) of the participants held a bachelor’s degree or higher. Self-reported body mass index (BMI) ranged from 13.1 to 64.8 (*M* = 24.5; SD = 7.8), with 18.7% of participants reporting a BMI > 18.5. Approximately half of the participants (51.6%) self-reported a current ED diagnosis by a health professional. An overview of demographic information is presented in Table 1. There was a significant difference in BMI between the participants included in the study and the non-completers (*M* = 25.5; SD = 8.2); however, there was no different in other demographic variables (i.e., age and sex; results not displayed).

**Table 1:** Means, standard deviations, and group comparisons of demographic information for participants in the EFA and CFA analyses

	Overall sample (*N* = 826)	EFA sub-sample 1 (*n* = 413)	CFA sub-sample 2 (*n* = 413)	Statistics = *p*
Age in years (*M/SD*)	26.8 (8.6)	26.3 (8.1)	27.3 (9.2)	0.100
Sex (*n*/%)				0.112
Men	60 (7.3%)	27 (6.5%)	33 (8.0%)	
Women	753 (91.2)	376 (91%)	377 (91.3%)	
Not specified	13 (1.6%)	10 (2.4%)	3 (0.7%)	
Height in m (*M/SD*)	1.7 (0.1)	1.7 (0.1)	1.7 (0.09)	0.664
Weight in kg (*M/SD*)	67.7 (22.7)	66.3 (21.5)	69.2 (23.8)	0.066
BMI (*M*/SD)	24.5 (7.8)	24.1 (7.8)	24.9 (7.9)	0.108
Education (*n*/%)				0.788
Did not complete grade 10	3 (0.4%)	0 (0.0%)	3 (0.7%)	
Grade 10	25 (3.0%)	10 (2.4%)	15 (3.6%)	
Grade 12	243 (29.4%)	125 (30.3%)	118 (28.6%)	
Vocation school or diploma	164 (19.9%)	78 (18.9%)	86 (20.8%)	
Bachelor’s degree	288 (34.9%)	152 (36.8%)	136 (32.9%)	
Master’s degree	82 (9.9%)	40 (9.7%)	42 (10.2%)	
PhD	21 (2.5%)	8 (1.9%)	13 (3.1%)	
Nationality (*n*/%)				0.157
American	424 (51.3%)	220 (53.3%)	204 (49.4%)	
Australian	124 (14.5%)	51 (12.3%)	69 (16.7%)	
British	77 (9.3%)	30 (7.3%)	47 (11.4%)	
Canadian	40 (4.8%)	20 (4.8%)	20 (4.8%)	
New Zealander	13 (1.6%)	6 (1.5%)	7 (1.7%)	
Other	152 (18.4%)	86 (20.8%)	66 (16.0%)	
Self-reported current ED diagnosis (*n*/%)				0.403
Yes	426 (51.6%)	219 (53.0%)	207 (50.1%)	
No	400 (48.4%)	194 (47.0%)	206 (49.9%)	

EFA: exploratory factor analysis; CFA: confirmatory factor analysis; BMI: body mass index

### Instruments

#### Demographics measures

Participants’ age, sex, weight, height, education, and nationality were recorded. Body mass index was calculated using the formula: weight (kilograms)/height^2^ (meters). Participants were asked whether they have received a current mental health diagnosis (e.g., depression, anxiety, eating disorder, etc.) or an ED diagnosis from a health professional, and to specify the diagnosis.

#### Young Positive Schema Questionnaire (YPSQ)

The YPSQ is a 56-item questionnaire [57] that aims to assess 14 early adaptive schemas. Items are rated on a 6-point scale ranging (1 = completely untrue of me, 6 = describes me perfectly), for example, ‘*for much of my life, I have felt that I am special to someone’, ‘I’m usually comfortable expressing my feelings to others when I want to’, and ‘I like to do well but don’t have to be the best’*. Higher scores indicate higher levels of EAS. The YPSQ has been found to have good internal consistency (i.e., α = 0.72–0.89, [63]. An error was made while formatting the YPSQ to the survey platform and the items were ordered as per their factors and not in the same order as the original questionnaire. Additionally, one item (item 55—‘When I ask someone for something and the answer is “no,” I’m usually comfortable accepting it without pushing to get my own way’) was left out of the survey, resulting in 55-items instead of 56.

#### The Eating Disorder Examination Questionnaire (EDE-Q)

The EDE-Q 6.0 [25] is a 28-item self-report measuring the severity of ED psychopathology. The questionnaire consists of four subscales (i.e., Restraint; Eating concern; Shape concern, and Weight concern) with each question measured on a 7-point scale (0–6). Subscale scores are calculated by adding the relevant subscale items and then dividing the sum of scores by the number of total items in the subscale. A global score is calculated by taking the average of the subscale scores (i.e., adding the subscales and dividing by the number of subscales). Higher scores indicate greater levels of ED pathology in the relevant domain. Additional behavioural items are used to measure frequency of ED related behaviours (e.g., binging, purging, laxatives use, excessive exercise, and food restriction) that are not included in the subscale total scores. These items were used to assist the classification of participants with significant ED symptoms to meet inclusion criteria for this study, as outlined in the Participant section. The EDE-Q subscales and full measure has been found to have good internal consistency (i.e., Global score: α = 0.90; Restraint: α = 0.70; Eating Concern: α = 0.73; Shape Concern: α = 0.83; Weight Concern: α = 0.72; [66]. Cronbach's alpha in the present study were as followed: Global score: α = 0.86; Restraint: α = 0.77; Eating Concern: α = 0.66; Shape Concern: α = 0.78; Weight Concern: α = 0.59.

#### Emotional Regulation Questionnaire (ERQ)

The ERQ [33] is a 10-item self-report measuring two facets of emotional regulation: Cognitive Reappraisal (e.g., *‘When I’m faced with a stressful situation, I make myself think about it in a way that helps me stay calm’*) and Expressive Submission (e.g., *‘I control my emotions by not expressing them’*. Each question is answered on a seven-point Likert scale, ranging from (1 strongly disagree to (7 strongly agree. Total scores for each independent subscale are calculated by summing relevant subscale scores. Higher scores on each scale indicate greater use of that coping strategy. Both subscales were shown to have good internal consistency in a population of individuals with EDs (i.e., Cognitive Reappraisal: α = 0.87; Expressive Suppression: α = 0.80; [17]. Internal consistency was similar in the present study also (i.e., Cognitive Reappraisal: α = 0.84; Expressive Suppression: α = 0.77).

#### Cognitive Flexibility Inventory (CFI)

The CFI [20] measures cognitive flexibility when faced with challenges. The inventory consists of 20-items which are measured on a seven-point Likert scale, ranging from (1) strongly disagree to (7) strongly agree. The CFI consists of two subscales, Alternate (e.g., *‘I like to look at difficult situations from many different angles’*) and Control (e.g., *‘I am capable of overcoming the difficulties in life that I face’*). Subscale scores are calculated by summing relevant subscale scores and a global score is cumulated by adding both sub scores. Higher scores indicate greater flexibility. The CFI subscales and full measure has been found to have good internal consistency (Alternate: α = 0.91; Control: α = 0.85; Global score: α = 0.90; [20], with similar scores found in the present study (i.e., Alternate: α = 0.91; Control: α = 0.86; Global score: α = 0.89).

### Procedure

Individuals over the age of 18-years were recruited through social networking sites (e.g., Facebook) and online forums (e.g., Reddit) via the distribution of a survey using the Qualtrics platform between September 2020 and May 2021. Advertisement of the survey was targeted at ED related pages and forums to increase the chance of capturing individuals with ED symptoms. The survey was completed online and took approximately 25 min to complete. A detailed information sheet including a description of the study and its aims was presented on the first page of the electronic survey. Individuals were informed that their data would be recorded anonymously and participation in the research was voluntary, and they would be able to exit the survey at any time. Consent was obtained via participants selecting a checkbox prior to beginning the survey. After the survey, participants were provided with contact details for support services, and they were also given the opportunity to enter a draw to win one of four $50 AUD Amazon vouchers which were drawn at the completion of the project. The study was approved by the University of the Sunshine Coast Ethics Committee (no. S201469).

### Statistical approach

The sample was randomly split into two groups using IBM SPSS Statistics 26 [44] random sample function. Exploratory factor analysis (EFA), using principal axis factoring, was used in half the sample (*n* = 413) to investigate the factor structure of the YPSQ. A confirmatory factor analysis (CFA) was conducted using the other half of the sample to confirm the factor structure of the EFA. Descriptive statistics, normality testing, and EFA was conducted with SPSS, and CFA was conducted using IBM SPSS Amos 26 [3].

Parallel analysis [42] was used to determine the number of factors to be used in further analyses. The paralleled analysis was set with the following criteria: Variables = 55, sample size = 413; number of random correlation matrices = 500; and percentile = 95; resulting in nine factors. The EFA, using principal axis analysis, was run with the number of factors fixed to nine. Promax rotation [40] was used to give a more accurate and realistic representation of the factors as items were expected to be correlated [23]. Further factor selection was based on methods from Louis et al. [57] and Paetsch et al. [63], such as the inclusion of factors with at least three items that all load above 0.40 and removal of items that have above loading above 0.40 on more than one factor. Pearson correlations were used to assess multicollinearity of YPSQ items [72] and correlation coefficients < 0.90 were deemed appropriate [36, 37].

A CFA was used to confirm the 48-item, nine-factor structure resulting from the EFA. Factors were labelled following Louis et al. [57] and Paetsch et al. [63]. The metric of each latent variable was determined by constraining their residual variance to 1. Model fit was determined using several indices: Root-Mean Square Error of Approximation (RMSEA [81], with ≤ 0.05 = good fit or ≤ 0.08 = acceptable fit [90% confidence intervals]; Standard Root Mean Square Residual (SRMR; [43]), with ≤ 0.05 = good fit or ≤ 0.08 = acceptable fit; Comparative Fit Index (CFI; [7], with ≥ 0.95 = good fit or ≥ 0.90 = acceptable fit; and Tucker–Lewis Index (TLI; [88], with ≥ 0.95 = good fit or ≥ 0.90 = acceptable fit. The “Jackknife” approach was used to improve model fit indices were not acceptable. This approach was also used by Louis et al. [57] and involves targeting items with low regression weight and/or high inter-item correlations for removal to improve model fit [48].

Internal consistency for all scales were assessed using Cronbach’s alpha coefficients with values of 0.7 considered acceptable [14]. Convergent validity of the YPSQ was testing using Pearson correlation with EDE-Q, ERQ, and CFI. Significance was determined at *p* < 0.05 level and effect size measured by positive or negative correlation coefficient (i.e., small =  < 0.30; medium = 0.30 to 0.50; and large =  > 0.05; [11].

## Results

### Normality testing

Frequency tests were run on both ED groups for EFA and CFA to assess normal distribution of demographic variables and YPSQ output. The distribution of all data was deemed normal with skewness ranging from -0.99 to 1.84 and kurtosis ranging from − 1.30 to 4.45 [8, 36, 37]. No significant between-groups differences were observed for any of the demographic variables (see Table 1).

### Exploratory factor analysis

An EFA was conducted in the first randomly selected half of the sample. The KMO = 0.90 and Bartlett’s test of sphericity was significant (*χ*^2^ = 14,517.78, *df* = 1485, *p* =  > 0.000), indicating the data was appropriate for EFA. All correlations were *r* =  < 0.90 suggesting no evidence of substantial multicollinearity. Factors were fixed to nine, as per the paralleled analysis, which explained 56.3% variance of the model. Four items were removed due to not loading on any factors; and three items were removed for cross loading (*r* =  > 0.40) on more than one factor. The EFA was run again after item removal, with a total of 48 items. The KMO = 0.89 and Bartlett’s test of sphericity was significant (*χ*^2^ = 12,467.04, *df* = 1128, *p* =  > 0.000), and 58.6% of the model variance was explained. All nine factors had at least three item loadings, while the first factor included 12 items. The average within factor correlation was *r* = 0.73 (range *r* = 0.56–0.83) and the average between factor correlation was *r* = 0.22 (range *r* = − 0.13–0.52; see Table 2 for factor loadings).

**Table 2:** Factor loading matrix

Items	Factor								
	1	2	3	4	5	6	7	8	9
YPSQ_2		0.493							
YPSQ_3		0.439							
YPSQ_4		0.532							
YPSQ_5		0.584							
YPSQ_6			0.707						
YPSQ_7			0.754						
YPSQ_8			0.851						
YPSQ_9			0.845						
YPSQ_10			0.876						
YPSQ_11							0.730		
YPSQ_12							0.760		
YPSQ_13							0.762		
YPSQ_14									0.488
YPSQ_15									0.442
YPSQ_17									0.709
YPSQ_18									0.583
YPSQ_19						0.653			
YPSQ_20						0.818			
YPSQ_21						0.780			
YPSQ_22						0.833			
YPSQ_23	0.654								
YPSQ_24	0.677								
YPSQ_25	0.608								
YPSQ_26								0.826	
YPSQ_27								0.982	
YPSQ_28								0.449	
YPSQ_29				0.743					
YPSQ_30				0.835					
YPSQ_31				0.841					
YPSQ_32				0.957					
YPSQ_33				0.786					
YPSQ_34					0.678				
YPSQ_35					0.759				
YPSQ_36					0.872				
YPSQ_37					0.820				
YPSQ_38	0.614								
YPSQ_39	0.733								
YPSQ_40	0.739								
YPSQ_41	0.701								
YPSQ_42	0.599								
YPSQ_43	0.538								
YPSQ_44	0.549								
YPSQ_45	0.533								
YPSQ_46	0.594								
YPSQ_49		0.791							
YPSQ_50		0.900							
YPSQ_51		0.863							
YPSQ_52		0.744							

### Confirmatory factor analysis

A 48-item, nine-factor structure was tested in AMOS as per the results from the EFA. Factors were labelled accordingly to Louis et al. [57] and Paetsch et al. [63]. The metric of each latent variable was determined by constraining their residual variance to one. The overall model showed good adequate fit, χ^2^(1047) = 3396.17, *p* = 0.000. The root mean square error of approximation (RMSEA; 0.074 [0.071, 0.071]) and standardised root mean squared (SRMR; 0.072) showed acceptable fit, however, comparative fit index (CFI; 0.816) and Tucker–Lewis index (TLI; 0.802) indices showed poor fit. The Jackknife approach [48] was used by removing items with low regression weights (< 0.600 [36, 37], and high inter-item correlations. Eight items were removed due to low regression weights and one item was removed due to high covariance with two other items. Finally, three pairs of items were co-varied due high correlated error residues and similar wording. The adjusted model had 39 items, contributing to nine factors. The CFA results for the final model showed acceptable fit, χ^2^ (666) = 1,380.68, *p* = 0.000, RMSEA (0.051 [0.047, 0.055]), SRMR (0.057), CFI (0.931), and TLI (0.923). Table 3 shows a comparison of EAS factors across Louis et al., Paetsch et al., and the current study.

**Table 3:** Comparison of the Early Adaptive Schema (EAS) Structure in Louise et al. [57], Paetsch et al. [63], and the Current Paper

Louis et al.—14 EAS factors	Paetsch et al.—10 EAS factors	Current Study—9 EAS factors
Emotional fulfillment	Emotional fulfillment	Emotional fulfillment and stable attachment^†^
Stable attachment	Stable attachment	
Success	Success	Success
Empathic consideration	Empathic consideration	Empathic consideration
Basic health and safety/optimism	Optimism	Optimism
Emotional openness and spontaneity	Emotional openness	Emotional openness
Self-compassion	Self-compassion and realistic expectations^†^	Self-compassion and realistic expectations^†^
Realistic expectations		
Healthy self-interest/self-care		Healthy self-interest/self-care^‡^
Healthy Boundaries/developed self	Developed self^†^	Developed self
Healthy self-reliance/competence		Healthy self-reliance/competence^‡^
Self-directedness	Self-directedness^‡^	Self-directedness^‡^
Social belonging	Social belonging	Social belonging
Healthy self control/self discipline	Healthy self-control	Healthy self-control

EAS: early adaptive schemas

^†^Merged factors; ^‡^Removed factors

### Validity

All nine factors showed good internal consistency with Cronbach’s alpha ranging between 0.77 (Developed Self) and 0.92 (Social Belonging; see Table 4). All EAS except for Empathic Consideration showed an inverse relationship to the EDE-Q Global score. Furthermore, all other EAS showed a similar significant relationship to all EDE-Q subscale, except for the EAS Developed Self, which only showed a significant inverse relationship to the Eating Concerns EDE-Q subscale, and Success, which failed to show a significant relationship to the Restraint EDE-Q subscale. One positive relationship was found between the EAS Healthy Self-Control and the Restraint EDE-Q subscale (see Table 5). All EAS showed a significant positive relationship to the CFI Total score and Alternate and Control subscales, except for the EAS Empathic Consideration and Control CFI subscale (*r* = 0.08–0.44). All EAS showed a significant positive relationship to the Cognitive Appraisal ERQ subscale (*r* = 0.10–0.38) and all EAS except Developed Self. Healthy Self-Control showed a significant inverse relationship to the Expressive Suppression ERQ subscale (*r* = 0.13–0.60; see Table 6).

**Table 4:** Means, standard deviations, and alphas for the new nine-factor model in the full ED sample

Early adaptive schemas	*M*	SD	α
Emotional fulfillment and stable attachment (6)	3.1	1.3	0.89
Success (5)	4.0	1.2	0.90
Empathic consideration (3)	4.3	1.1	0.80
Optimism (4)	2.7	1.1	0.83
Emotional openness (4)	3.3	1.3	0.87
Self-compassion and realistic expectations (6)	2.4	1.0	0.87
Developed self (3)	4.2	1.4	0.77
Social belonging (5)	2.2	1.0	0.92
Healthy self-control (4)	3.5	1.3	0.87

The number of items per factor/EMS is displayed in parentheses

**Table 5:** Pearson’s correlations coefficients between EAS and EDE-Q in the full ED sample

Early adaptive schemas	EDE-Q Restraint (*n* = 826)	EDE-Q eating concerns (*n* = 826)	EDE-Q shape concerns (*n* = 826)	EDE-Q weight concerns (*n* = 826)	EDE-Q global score (*n* = 826)
Emotional fulfillment and stable attachment	− 0.24**	− 0.28**	− 0.33**	− 0.29**	− 0.36**
Success	− 0.04	− 0.21**	− 0.20**	− 0.16**	− 0.19**
Empathic consideration	− 0.04	− 0.02	− 0.01	0.02	− 0.02
Optimism	-0.20**	− 0.28**	− 0.33**	− 0.29**	− 0.34**
Emotional openness	− 0.19**	− 0.16**	− 0.18**	− 0.11*	− 0.21**
Self-compassion and realistic expectations	− 0.28**	− 0.34**	− 0.38**	− 0.32**	− 0.42**
Developed self	− 0.05	− 0.17**	− 0.07	− 0.06	− 0.11*
Social belonging	− 0.13**	− 0.21**	− 0.31**	− 0.21**	− 0.26**
Healthy self-control	0.07*	− 0.17**	− 0.16**	− 0.13**	− 0.11*

EDE-Q: Eating Disorder Examination Questionnaire

^*^p < 0.050. **p < 0.001. *r* values ≥ 0.30 are bolded

**Table 6:** Pearson’s correlation coefficients between EAS and ERQ, and CFI in the Full ED sample

Early adaptive schemas	ERQ cognitive reappraisal (*n* = 791)	ERQ expressive submission (*n* = 791)	CFI alternate (*n* = 808)	CFI control (*n* = 808)	CFI total (*n* = 808)
Emotional fulfillment and stable attachment	0.26**	− 0.26**	0.18**	0.35**	0.32**
Success	0.25**	− 0.11**	0.31**	0.42**	0.44**
Empathic consideration	0.10**	0.13**	0.23**	0.04	0.18**
Optimism	0.32**	− 0.18**	0.12**	0.39**	0.30**
Emotional openness	0.13**	− 0.60**	0.17**	0.23**	0.25**
Self-compassion and realistic expectations	0.38**	− 0.20**	0.08*	0.40**	0.27**
Developed self	0.13**	− 0.05	0.12**	0.22**	0.20**
Social belonging	0.26**	− 0.28**	0.17**	0.37**	0.32**
Healthy self-control	0.26**	0.05	0.29**	0.33**	0.38**

ERQ: Emotional Regulation Questionnaire; CFI: Cognitive Flexibility Inventory

^*^p < 0.050. **p < 0.001. *r* values ≥ 0.30 are bolded

## Discussion

The present study explored the psychometric properties of the YPSQ in a population of individuals with ED symptomatology and investigated the relationship between EAS, ED symptomatology, emotional regulation (i.e., cognitive reappraisal and expressive suppression), and cognitive flexibility. The first hypothesis was supported, finding a shorter, 39-item, nine-factor model compared to the original 56-item, 14-factor structure from Louis et al. [57] and the 41-item, 10-factor structure from Paetsch et al. [63]. The nine-factor model showed acceptable model fit and psychometric properties. The second hypothesis was mostly supported, finding all but one EAS (i.e., Empathic Consideration) showed a significant inverse relationship with ED symptomatology. All EAS showed a significant positive relationship with cognitive flexibility and cognitive reappraisal. All but one EAS (i.e., Developed Self) showed a significant inverse relationship with expressive suppression.

The 14-factor structure retained seven EAS from both validation studies by Louis et al. [57] and Paetsch et al.[63], (see Table 3 for comparison of EAS structure of Louise et al., Paetsch et al., and the current paper). Four of the original EAS were collapsed into two factors and three original EAS were not included due to either poor loading on the EFA or low regressions weights.

The inconsistency of EAS found in each study could be explained by the different samples used. The current study used a sample of mostly women with ED symptomatology which may have restricted the variance and limited the number of unique factors from emerging [10, 69]. Individuals with EDs show higher levels of all EMS, except Entitlement, when compared to community samples, [21, 46, 50, 58, 89, 90], suggesting they would also show lower levels of EAS compared to a community sample. If this is the case, item scores are likely to group together at the lower end of the scale, concealing a more complex factor structure that may have been achieved from a sample of individuals with more varied positive beliefs about themselves. However, the primary EFA sample used in the study by Paetsch et al. [63] was drawn from a community sample and showed dissolution of the same factors in the current study, albeit, only the EAS Self-Directedness was removed completely and the other factors were merged (see Table 3). This conflicts with idea that the ED characteristics of the sample were responsible for the dropped factors in the current study.

The sample used in Paetsch’s et al. EFA comprised of ~ 80% women, which was similar to the characteristics of the current study (i.e., ~ 90% women), and differed to the proportion of women used in the original validation paper by Louis et al. [57], i.e., ~ 54% women). As this is the only noticeable homogenising characteristic in both follow-up validation studies that differ to the original study by Louis et al., it suggests that there may be a sex difference in EAS variance between men and women. No research to the authors knowledge has explored this, however, research on EMS differences between men and women has shown varied results. A paper by Shorey et al. [77], found that all EMS except Entitlement, Emotion Inhibition, Unrelenting Standards, and Punitiveness were significantly greater in a sample of women diagnosed with alcohol dependency disorder compared men with the same diagnosis. Tremblay and Dozois [87] found alternate results in a sample of university students, with men having higher Emotional Deprivation and Entitlement, but lower Self-Sacrifice EMS compared to women. Future studies are needed to determine whether such EAS sex differences exist.

The second hypothesis was mostly confirmed, finding significant correlations between the YPSQ and the EDE-Q, CFI, and ERQ, demonstrating good convergent validity. The relationship between EAS and ED symptoms, measured by the EDE-Q subscales, mostly showed significant weak inverse relationships, except for the EAS Empathic Consideration which was not significant.

The EDE-Q Shape Concerns subscale showed the strongest inverse relationships with most EAS compared to the other EDE-Q subscales, with the strongest effect sizes for Self-Compassion and Realistic Expectations, Emotional Fulfilment and Stable Attachment, Optimism, and Social Belonging. The EDE-Q Restraint subscale showed only weak inverse relationships to EAS and contained the only significant positive correlation being the Healthy Self-Control EAS. These results suggests that the absence of positive beliefs about one’s body image and eating behaviour, particularity one’s body shape, is a driving factor for EDs, which is congruent with Fairburn’s et al. [27] transdiagnostic theory.

These findings are also supported by a recent 2021 systematic review by Maher et al. [58] exploring EMS in ED symptoms, which found the EMS Unrelenting Standards, the counterpart to the EAS Self-Compassion and Realistic Expectations, was pervasive across all ED diagnoses and had the highest grand mean when compared with all other EMS. This is further supported by research finding that perfectionism [6] and low self-compassion [29] are a common component of EDs, which demonstrates the need for interventions that focus on self-compassion and realistic expectations [53]. Maher et al. also found the EMS Emotional Deprivation and Abandonment, the counterparts to the EAS Emotional Fulfilment and Stable Attachment, were strongly associated with binge eating disorder presentation. The EMS Negativity/Pessimism, the counterpart to the EAS Optimism, was not found to be associated with ED presentations in the Maher et al. review; however, other research has shown higher Negativity/Pessimism scores in individuals with ED pathology compared to control groups [15, 21]. The term ‘counterpart’ is used tentatively as the relationship between EMS and EAS is yet to be explored in detail and is likely to be more complex than the two ends of a bipolar scale [57].

The Empathic Consideration was the only EAS that did not have a significant inverse relationship to the EDE-Q. Entitlement, the EMS counterpart to Empathic Consideration, has been shown to be the least likely EMS to be significantly higher in individuals with ED pathology when compared with healthy controls [58]. Furthermore, Paetsch et al. [63] failed to find a significant difference in Empathic Consideration between a community sample and psychiatric sample. Narcissism (i.e., Entitlement) is not associated [55] or weakly associated with ED pathology [92]. Waller et al. [92] found that narcissism in individuals with EDs was characterised by a tendency of putting others needs before one’s own (i.e., Subjugation EMS) resulting in feeling mistreated and abused by others (i.e., Mistrust/Abuse EMS), described as a ‘poor me’ narcissistic defence of self-esteem. Therefore, Entitlement EMS and Empathic Consideration EAS may be expressed as by Subjugation related beliefs, which could explain why it was not significantly correlated with EDE-Q scores. Further research is needed to test this.

All EAS except Empathic Consideration showed significant positive relationships with the total score and both subscales of the CFI. The Control subscale showed a stronger relationship to EAS than the Alternate subscale, with all but three EAS (e.g., Empathic Consideration, Emotional Openness, and Developed Self) having medium effect sizes. This is theoretically supported, as the EAS with medium effect sizes share aspects of motivation and control which is a similar construct being measured in the Control subscale (e.g., perceived controllability of one’s environment). Furthermore, high Control scores are inversely related to self-blame and wishful thinking [20], which are common cognitive strategies used by individuals with EDs [56, 64]. The EAS Optimism, and Self-Compassion and Realistic Expectations showed the strongest positive relationship to the Cognitive Reappraisal ERQ subscale, while the EAS Emotional Openness had the strongest inverse relationship to the Emotional Suppression subscale.

### Limitations

As stated in the method section, an error was made while formatting the YPSQ, resulting in a different ordering of the survey items and missing item 55. A decision was made to continue the factor analyses as the factor from which the item was missing (i.e., Empathic Consideration) still contained three items, indicating an acceptable representation of latent constructs, ‘particularly when other constructs have more than three [items]’ [36, 37], pp. 610–611). Furthermore, Guadagnoli and Velicer [34] showed that sample size and construct saturation are the most important factors contributing to factor stability, with even weakly saturated components (i.e., *r* = 0.40) or low items/construct ratio providing stable solutions with sample sizes between 300 and 400.

It is unknown how these errors have impacted the results, such as, whether the ordering of items influenced participants to answer in a biased way, or whether a different factor structure would have emerged. However, the results suggest that these errors may be minimal or absent as half of the factors remained the same as Louis et al. [57] and Paetsch et al. [63] and the modifications to the factor structure that were made closely corresponded to Paetsch et al. Furthermore, the missing item from Empathic Consideration did not seem to impact the factor loading in the current study, nor did it seem to influence the internal validity of the factor. The missing item remained in the paper by Paetsch et al., making Empathic Consideration a four-item factor with an α of 0.70 in the community sample and 0.77 in the psychiatric patient sample. The internal consistency of the three-item Empathic Consideration factor in the present study had an α of 0.80.

A third limitation was that the ED sample was identified using self-report measures and may not accurately reflect individuals with a diagnosed ED, thus, caution should be taken if generalising the results to individuals with ED diagnosis. Fourth, the sample primarily consisted of women, which limits the generalisability of the results to men and may have influenced the variability of the EAS factor structure. Under-diagnosis and underrepresentation of men with EDs has been previously documented [30, 82, 93], which aligns with the current sample. Finally, the study lacked additional validation measures (e.g., YSQ) for a more thorough investigation of the construct validity and discriminant validity of the YPSQ.

### Clinical implications

The current study has further demonstrated the relationship between schemas and ED symptomology, supporting the beneficial role of schema theory in contributing to a more thorough conceptualisation and understanding of ED pathology. The nine-factor YPSQ could be useful in a clinical setting for case conceptualisation and to help develop targeted treatment plans, by identifying and addressing low scoring EAS of individuals with ED pathology. For example, strength-based interventions addressing low self-compassion and Realistic Expectations EAS could focus on building self-compassion and self-esteem, through increasing cognitive flexibility healthy coping strategies. Further research is needed to confirm the factor structure of the nine-factor YPSQ in other clinical populations and its relationship to the YSQ. For instance, exploring the unique combinations of both EMS and EAS in different populations.

### What is already known on this subject

Research has shown that individuals with EDs often present with higher levels of maladaptive schemas compared to the general population. Recent research has explored the profile of adaptive schemas in general and clinical populations; however, EAS profile has not been assessed in a population of individuals with EDs. Exploration of EAS in individuals with ED symptomatology may inform further development of schema-based, positive psychology, therapies.

### What this study adds

This study demonstrated a unique nine-factor YPSQ with good internal validity in a population of individuals with ED symptomatology. The nine-factor YPSQ could be used in a clinical setting to assist in identifying EAS deficits in individuals with EDs to develop tailored therapy to address these, such as compassion-based therapy to increase self-compassion and realistic expectations. Further research is needed to expand these results and generalise the nine-factor YPSQ to other clinical populations.

## Data Availability

The dataset used and analysed during the current study are available from the corresponding author upon reasonable request.
